# Deaths from homicides: a historical series^1^

**DOI:** 10.1590/0104-1169.3603.2511

**Published:** 2014

**Authors:** Flávia Azevedo de Mattos Moura Costa, Ruth França Cizino da Trindade, Claudia Benedita dos Santos

**Affiliations:** 2 PhD, Assistant Professor, Departamento de Ciências da Saúde, Universidade Estadual de Santa Cruz, Ilhéus, BA, Brazil; 3 PhD, Associate Professor, Universidade Federal de Alagoas, Maceió, AL, Brazil; 4 PhD, Associate Professor, Escola de Enfermagem de Ribeirão Preto, Universidade de São Paulo, WHO Collaborating Centre for Nursing Research Development, Ribeirão Preto, SP, Brazil

**Keywords:** Homicide, Mortality, Violence

## Abstract

**OBJECTIVE:**

to describe mortality from homicides in Itabuna, in the State of Bahia.

**METHOD:**

study with hybrid, ecological and time-trend design. The mortality coefficients per 1,000 inhabitants, adjusted by the direct technique, proportional mortality by sex and age range, and Potential Years of Life Lost were all calculated.

**RESULTS:**

since 2005, the external causes have moved from third to second most-common cause of death, with homicides being responsible for the increase. In the 13 years analyzed, homicides have risen 203%, with 94% of these deaths occurring among the male population. Within this group, the growth occurred mainly in the age range from 15 to 29 years of age. It was ascertained that 83% of the deaths were caused by firearms; 57.2% occurred in public thoroughfares; and 98.4% in the urban zone. In 2012, the 173 homicides resulted in 7,837 potential years of life lost, with each death causing, on average, the loss of 45.3 years.

**CONCLUSIONS:**

mortality by homicide in a medium-sized city in Bahia reaches levels observed in the big cities of Brazil in the 1980s, evidencing that the phenomenon of criminality - formerly predominant only in the big urban centers - is advancing into the rural area of Brazil, causing changes in the map of violent homicide in Brazil.

## Introduction

Characterized as a phenomenon with complex and multifactorial causality, one can define violence as actions undertaken by one or more individuals and which cause physical or psychological harm to oneself or to others^(^ 1 ^)^. In this regard, it is deeply rooted in the social, economic and political structures, representing a risk for the process of human development, with potential threats to life and to health and the consequent possibility of death^(^ 2 ^)^.

Among the various forms of expression of violence, homicide is the most outrageous act, as it "definitively deprives the victim of all her rights", thus being an indicator for society's inability to develop and maintain non-lethal mechanisms for conflict resolution. Attention is drawn to mortality from homicides, fundamentally, because, in addition to occurring in large numbers, it mainly affects a young population. It is the principal cause of death in the age group between 15 and 44 years old, subverting the pattern present in first world countries, which is that deaths occur at the more advanced ages, being the main cause of potential years of life lost in this population^(^ 1 ^-^ 3 ^)^.

In Brazil, since the last decade, the concentration of homicides which previously was present in the major cities has spread to the interior of the country, as organized crime has sought new spaces. Besides the public safety institutions' difficulties in containing the process of the spread of violence into the interior of the country, urban degradation has contributed decisively to it, as poverty, social inequality, and poor access among the population to goods and basic services are problems which are no longer exclusive to the big cities^(^ 1 ^)^.

Research undertaken by the Ministry of Justice (MJ) and the NGO the Brazilian Forum on Public Safety, involving 266 municipalities with more than 100,000 inhabitants, in 2009, confirms that although it is spread throughout Brazil, violence is growing in the North and Northeast. This is a reflection of poor social indicators, few resources for applying to the public safety systems, and few preventive policies^(^ 4 ^)^.

It is in Bahia that one finds five of the 15 municipalities indicated by the above-mentioned study undertaken by the MJ, where the young Brazilians are most exposed to criminality, according to the Youth Vulnerability to Violence Index (IVJ-Violence) which takes into account socioeconomic data such as the number of assaults, educational level, access to the job market, income and housing^(^ 4 ^)^.

The most serious situation is that of the municipality of Itabuna, which occupies first place in the research's general ranking, evidencing that young males are increasingly involved as victims and authors of deaths by homicide. These deaths, in the urban spaces, are linked to impunity for infractions of the law and delinquency; to the exaggerated consumption of alcoholic drinks; to the use of, and trafficking in, drugs; to wide access to, and availability of, firearms; and to the absence of a political project for greater inclusion, which could be capable of reducing the social exclusion to which various segments of society are subject^(^ 1 ^)^.

This study aims to describe mortality from homicides in Itabuna-Bahia in the period 2000 - 2012. It is believed that knowledge of the scale, characterization and tendencies of mortality from violent causes, in particular from homicides, in Itabuna, with emphasis on its distribution by sex, age, causes and time-trends in the period 2000 - 2012, in addition to the Potential Years of Life Lost for the year 2012, could support planning and implementation of actions, geared towards the area's specific characteristics, which could be effective in the reduction and prevention of these events, with a view to a greater positive impact on the population's levels of health and living conditions. 

## Method

A study with a hybrid, ecological and time-trend design (retrospective and longitudinal) was undertaken, focusing on mortality from homicide in the period 2000 - 2012, in the municipality of Itabuna, located in the South Region of Bahia, in the Cacaueira micro-region. For many years, this municipality's economic basis was the cultivation of cocoa beans. Since the end of the 1980s, it has been facing a serious crisis due to the appearance of witch's broom disease*, which caused a marked drop in production. Currently, the city is seeking economic alternatives, with the help of commerce, industry, and the diversification of plantations, and is an important commercial hub for the State, being sited on the edges of the BR-101 and BR-415 intercity highways^(^ 5 ^)^.

The population of the city of Itabuna was measured by a census in 2010 at 204,667 inhabitants, of whom 47.36% were men and 52.64% women; 97.54% lived in the urban zone^(^ 6 ^)^. The municipality has the fifth highest demographic density in the State of Bahia, with 464.54 inhabitants/km², and is made up of 59 neighborhoods, which are heterogenous in terms of demographic density, and which are marked by social inequality.

The study population covered the total number of homicides of persons living in Itabuna, and which occurred in the same municipality, in the period 2000 - 2012, obtained from the Mortality Information System (SIM), run by the Ministry of Health. The information was analyzed in terms of mortality coefficients (/1,000 inhabitants) and proportional mortality (/100)^(^ 7 ^)^, by sex and age range. The proportion of deaths by homicide was calculated regarding the total of deaths from external causes. Also used were census data provided by the Brazilian Institute of Geography and Statistics Foundation (IBGE), based on the censuses undertaken in 2000 and 2010, and the intercensus projections made available by the DATASUS*. 

In order to allow the comparison of the specific mortality coefficients by cause of death throughout the period under study, the researchers used the standardization of ages by the direct technique, with the aim of controlling for the effects of the changes in the age structure of the population over time. In this study, the population from the 2010 Census of the municipality of Itabuna in the State of Bahia (BA) was used as the standard population^(^ 7 ^)^.

The specific mortality coefficients by age range were applied in relation to the respective populational contingents of the standard population. The number of deaths anticipated which could occur in each age range was obtained, should the standard population be exposed to the specific mortality coefficients which, divided by the standard population, resulted in the Mortality Coefficient by Standardized Cause^(^ 7 ^)^.

Considering that standardization for comparison effect is not appropriate for the age-specific Mortality Coefficients, they were compared in their raw state.

In order to undertake the calculations of the Potential Years of Life Lost (PYLL) the technique proposed by Romeder and McWhinnie^(8) ^was used - PYLL between 1 and 70 years. In this calculation, only deaths which occurred between the ages of 1 and 69 complete years were considered. 

The formula for the calculation of the PYLL was undertaken in accordance with the expression: 



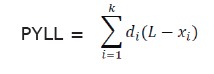



L = limit on length of life (only the deaths with ages below L will be considered);

x_i_ = age at which death occurred, with x_i_ < L;

d_i_ = represents the number of deaths with age x_i_i, 1 = K = L-1 in a population and in a given interval of time.

For the calculation of the PYLL for all external causes (ICD-10) with an age of 1 to 69 years (L=70), in the municipality of Itabuna, 2012, the decision was made to use the exact age at which each death occurred, subtracting it from L. The sum of these products provides the total PYLL, a value which represents the estimated number of losses for a specific cause of death or for all the causes.

As homicides, the following were considered: injuries caused intentionally, classified by the Tenth International Classification of Diseases (ICD-10), as "Assault" (X85 to Y09) as well as "Legal Intervention" (Y35 to Y36): they include assaults using firearms (X93-X95), assaults with edged weapons (X99) and other acts of violence. Legal Interventions (Y35) include trauma inflicted by the police or other representatives of the law, including those inflicted by on-duty members of the military and those which happen during the arrest or imprisonment, or attempts to do so, of lawbreakers, repression of rebellions, in maintaining order, and other legal actions^(^ 9 ^)^.

This study is part of the study termed "Spatial patterns of homicides associated with the Adapted Indicator for Living Conditions in the municipality of Itabuna - Bahia", approved by the Research Ethics Committee of the Ribeirão Preto School of Nursing, of the University of São Paulo, under protocol CAAE: 10176413.0.0000.5393.

## Results

In the period 2000 - 2012, 18,922 deaths were recorded on the SIM in Itabuna, of which approximately 10% (1,916 deaths) were from "symptoms, signs and abnormal findings from clinical tests, not classified elsewhere" and express the deaths for which there was no definition of basic cause.

**Figure 1 -:**
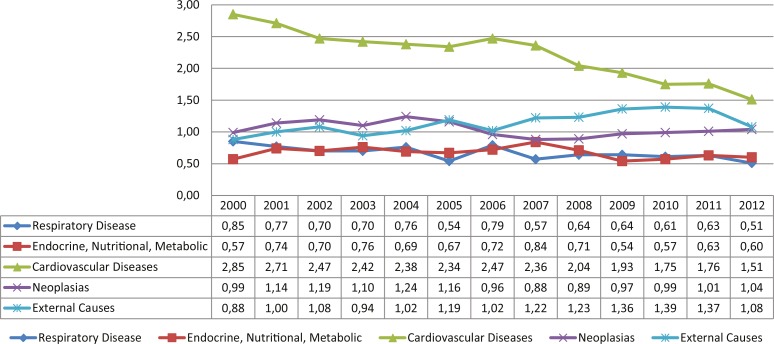
Distribution of the Mortality Coefficients (per 1000 inhabitants) standardized by age, using the direct technique, in accordance with five principal specific causes of death and year. Itabuna, BA, Brazil, 2000-2012

Of the deaths by defined causes, from 2005 onward, in the municipality of Itabuna, the external causes (accidents and violence), which occupied third place, moved to second place, as shown in Figure 1, which shows the mortality coefficients by specific cause, standardized by age, by the direct technique, in accordance with the following age ranges: less than one year; 1 to 4 years; 5 to 9 years; 10 to 14 years; 15 to 19 years; 20 to 29 years; 30 to 39 years; 40 to 49 years; 50 to 59 years, 60 to 69 years; 70 to 79 years and 80 years and over. Thus, in the period studied, the lowest coefficient of deaths from external causes was of 0.88/1,000 inhabitants in 2000, and the highest was 1.39/1,000 inhabitants in 2010, which represents a growth of approximately 37% of deaths from this set of causes. For the standardization of these coefficients by the direct technique, those deaths for which the age is unknown were discarded: 05 deaths from Cardiovascular Diseases; 02 deaths from Respiratory Diseases; 01 death from Neoplasia and 36 deaths from External Causes. 

When one examines the distribution of the total of 2973 deaths from external causes, which took place in the period 2000 - 2012, by specific types, it may be verified that 237 deaths, 8%, were recorded as "events in which the intention is unspecified" - that is, the information available was not sufficient to allow the distinction between it being an accident, a suicide, or a homicide. 

In relation to proportional mortality, in 2012, homicides were responsible for 82.5% of deaths from external causes for which the intention was defined, presenting considerable growth since 2000, when they corresponded to 38.6% of deaths from these causes. 

**Figure 2 -:**
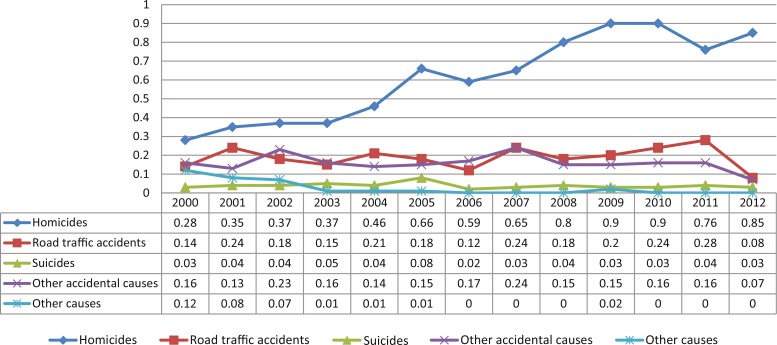
Distribution of the Mortality Coefficients (per 1000 inhabitants) of all ages, according to the set of external causes and year. Itabuna, BA, Brazil, 2000-2012

Figure 2 shows the growth of the coefficients of mortality from homicide, which from the beginning of the series, through to the year 2012, went from 0.28/1000 inhabitants to 0.85/1000 inhabitants. The other external causes (road traffic accidents, suicides, other accidental causes and other external causes involving medical and surgical complications) maintained stable coefficients until the year 2011, presenting a fall in 2012, which may indicate shortcomings in the recording of information. 

Observing the tendency of this set of causes by sex, clear differences may be observed, as approximately 95% of the deaths by homicide are concentrated in the male population. The increase of the coefficients of mortality from external causes for the men is owed, basically, to the increase in homicides, which from the year 2000 became three times higher, rising from 0.56 to 1.74 deaths per 1000 inhabitants in 2012. For the women, the coefficients varied from 0.03/1000 inhabitants in 2000 to 0.14/1000 inhabitants in 2011, declining to 0.05 in 2012. 

The scale of the proportional mortality from homicide among men, when compared to that of women, draws attention: the proportional mortality of the homicides among the men is equivalent to, on average, 15.3 times that found among the women, for the period in question. 

In Itabuna, in the period studied, an average of eight (8) in every 100 deaths of men were as a consequence of homicides, while for the women, only 0.5 were caused by homicides. 

**Figure 3 -:**
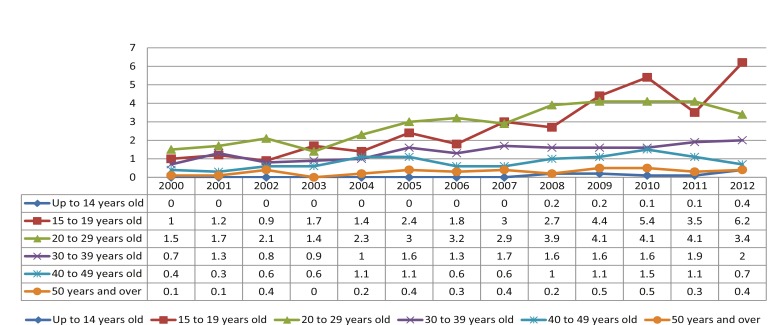
Distribution of the Coefficients of Mortality by homicides, male sex, by age groups and year. Itabuna, BA, Brazil, 2000-2012

The comparison, over the years studied, of the data relative to the coefficients of mortality by homicides, showed that among men increases occurred in all of the age ranges, with emphasis on that of 15 to 29 years old, in which the coefficients went from 1.0 to 6.2 deaths per 1000 inhabitants (Figure 3). Among women, the biggest coefficients were also recorded in this age range, varying from 0.08 in 2000, to 0.11 in 2012. In the other age ranges, among the women, the mortality coefficients fell. The percentage of deaths from homicide among men aged between 15 and 29 years old was 56 times greater than that recorded among the women in the same age group in 2012. 

The Potential Years of Life Lost (PYLL) of the population of Itabuna were calculated for the year 2012, as the year presented the highest coefficients of mortality from homicide in the population aged between 15 and 19 years old (coefficients of 3.1 for the general population and 6.2 for men). The homicides were responsible for 173 deaths, causing 7,837 potential years of life lost, each death causing, on average, the loss of 45.3 years (PYLL). Considering the limit of life to be equal to 70 years, it is estimated that, on average, the deaths occurred at the age of 24.7 years old (70.0 - 45.3 = 24.7). Homicides occupy the first place both in the number of cases and in PYLL, thus revealing early mortality as an important parameter in the measuring of a population's health conditions. 

Firearms were the weapon used most in homicides, in the period studied: 83% (1354/1634). Among men, firearms were used in the proportion of 83.6% (1288/1540) of the assaults; and in 70.2% (66/94) of the women affected. Blunt/edged weapons were responsible for the deaths of 24.5% (23/94) of the women and 8.9% (137/1540) of the men. The use of firearms in homicides grew 13% since the beginning of the decade beginning in 2000, coming to be responsible for 95% of the deaths in 2012. 

Of the total of deaths in which the intention was unknown, that is, unspecified (suicide, homicide or accident), 50.6% (78/154) were caused by firearms. However, in this group of deaths, this instrument had a significant fall over the period studied: between the years 2000 and 2006, it corresponded on average to 60%; coming, between 2007 and 2012, to correspond to 7.3% of the deaths in which the intention was unknown. 

It was ascertained that 3.7% (60/1634) of the deaths by homicide occurred in an unknown location. In those cases in which the place where the death occurred was identified, it was ascertained that 57.2% (901/1574) of the homicides occurred in public thoroughfares; 20.5% (322/1574) in hospital; 11.9% (187/1574) in another place and 10.4% (164/1574) in the home. The homicides in the urban zone corresponded to 98.4% (1548/1574), and in the rural zone, to 1.6% (26/1574). Deaths from homicide occurred in all the 59 neighborhoods of the city of Itabuna, with more than half of the crimes which occurred in homes and public thoroughfares being concentrated in 12 of these neighborhoods: 52.6% (560/1065).

**Table 1 -:** Distribution of the deaths - numerically, by percentage, and by indicators, according to basic cause. Itabuna, BA, Brazil, 2012

Basic cause (ICD-10)	N. of deaths	Death (%)	Mean age	Total of Potential Years of Life Lost	Potential Years of Life Lost (%)	Potential Years of Life Lost/Death
Homicides	173	82.0	24.7	7,837	89.2	45.3
Road traffic accidents	15	7.1	45.8	363	4.1	24.2
Other accidental injuries	11	5.2	42.9	298	3.4	28.1
Suicides	6	2.8	39.7	182	2.1	30.3
Unknown intention	5	2.4	51.4	93	1.1	18.6
Medical complications	1	0.5	61	9	0.1	9
All external causes	211	100	28.4	8,782	100	41.7

## Discussion

In collating the results referring to the structure of the cause of death, attention is drawn to the fact that external causes, in Itabuna, since 2005, have risen to second position, reaching levels which are comparable with those seen in the more developed regions of Brazil in the decades of the 1980s and 1990s, when the coefficients began to fall^(^ 10 ^)^. Studies indicate that in the year 2009, external causes represented the third most frequent cause of death in Brazil. However, this position was not uniformly distributed: the external causes were the second most frequent cause of death in the North, Northeast, and Center-West Regions; the fourth, in the Southeast Region, and the third, in the South Region. 

These results, in a certain way, may reflect on the one hand, the beginning of greater control of the violence in the regions which had already reached higher levels of their incidence, as is the case in the Southeast; on the other hand, they may indicate a process of the generalization of the violence to other areas. This study's data concur with the pattern of distribution observed by other authors in Brazil^(^ 1 ^,^ 3 ^)^, indicating that these health issues do not affect the population uniformly - that there are populational groups which are more vulnerable - which can be perceived by the unequal distribution of the deaths from external causes, which constitute the first cause of death among men aged between 10 and 39 years old. This distribution makes the problem more worrying, due to the fact that the population of adolescents and young people is the most common victim of the violence, placing the gains obtained in Brazilian life expectancy in recent years at risk. 

The data for mortality from homicides in Itabuna, in recent years, find a parallel with data from research undertaken in 1999 in São Paulo, the biggest metropolis in Brazil, with an estimated population of 9,968,485 inhabitants^(^ 11 ^)^, which corroborates the process of the spread of violence to the interior (i.e. areas and cities in rural regions) of Brazil and evidences that the shortcomings and inadequacies of the State and Public Safety Apparatus contribute to the attraction of criminality.

The predominance of mortality from homicide among young men found in this study was also observed in various other locales in Brazil. Some studies^(^ 1 ^,^ 12 ^)^ relate the excess mortality among males to the higher probability of exposure to violence. In this population, the growth of the numbers occurred in all the age ranges; however, the results from Itabuna indicate spread of the violence among an increasingly-young population: 647% among the men aged between 15 and 19 years old, in the period 2000 - 2012. Data published by the Ministry of Health for the year 2010 indicate the age range of 20 - 29 years old as being that at the highest risk of death from homicide in Brazil. One study on mortality from firearms observed that, in the period 1980 - 2010, there was an increase of homicides of 502.8% in the total population, and of 591.5% among the youngest (15 to 29 years old)^(^ 13 ^)^.

Authors^(^ 1 ^,^ 11 ^)^ contend that homicide of young people is related to the scarcity of protective factors and to areas where there is a large concentration of people in this age range. The victimization of increasingly young people is articulated with juvenile criminality, the recruiting of young people by drug trafficking, dropping out of school and gangsterism, all of which are mediated by the inability of the public social work bodies and the legal and police apparatus, within the context of social, institutional and family breakdown. 

These early losses of life bring harm not only to the individual and to the group who directly co-existed with him, but to the community as a whole, which is deprived of his economic and intellectual potential. In countries considered to be more developed (the United Kingdom, Canada and Japan, for example) generally speaking the structure of violent deaths is mainly composed of non-intentional elements, road traffic accidents and falls, exactly the opposite of what is observed for Brazil in general and the Municipality of Itabuna in particular. Even among the intentional elements, it is suicides which head mortality in these countries^(^ 3 ^)^.

Considering the indicator of potential years of life lost, mortality from homicides, in the year 2012, was shown in a concerning way in that it principally affected young adults: 45.3 PYLL per death, occurring, on average, at the age of 24.7 years old. The losses in productivity due to premature death or possible sequelae resulting from the violence are considerable. One study undertaken in Salvador, between 1998 and 2003, shows that the indicator corresponded to 42.4 PYLL per homicide, occurring, on average, at the age of 27.6 years old^(^ 14 ^)^.

Regarding the growing volume of studies focusing on the incidence of deaths from violent causes in Brazil, those which translate this phenomenon in terms of the estimated years of life lost as a result of these deaths remain scarce. This information, however, is important to sensitize those who formulate public policies to the need to direct actions with a view to reducing deaths from homicides. 

In Itabuna, it was observed that the male/female ratio among adolescents and young adults (56/01) is greater than that found for Brazil in 2009, which was 13/01^(^ 3 ^)^. The firearm was the weapon used most in the violent events which occurred in the municipality, for both sexes, a fact also observed by various authors^(^ 1 ^,^ 3 ^)^. A recent study has demonstrated that Alagoas, Bahia, Ceará, Pará and Paraíba were the States which presented the highest rates of homicide by firearm in the year 2010^(^ 13 ^)^.

The growth of 13% of the number of homicides with firearms is slightly superior to the global growth in Brazil, which was 11.2% in the decade 2000 - 2010^(^ 13 ^)^.These data contrast with those published by the Brazilian Institute for Applied Economic Research (IPEA)^(^ 14 ^)^, in which there was a fall of 12.6% in the rate of homicides in Brazil after 2003, with the creation of the Disarmament Statute (Law 10,826/03) which authorizes the carrying of guns by police officers, firefighters, gun collectors, and private security guards - and which prohibits this for other civilians).

Although the above-mentioned Law has been in place since 2004, it is estimated that in Brazil, at the present time, there is a vast arsenal of firearms in the hands of the population: 15.2 million, of which 6.8 million are registered and 8.5 million are not. Among these, 3.8 million are in the hands of criminals^(^ 15 ^)^. This issue of widespread use of, and ease of acquiring, firearms is disturbing for the whole of society. Newscasts on the communication networks speak about heavy weapons used by criminal networks, frightening the citizens who watch them. 

The occurrence of approximately 80% of the homicides in the place where the victim was attacked (public thoroughfare, the home and other public places such as bars, prisons and garbage dumps*) may indicate the intentionality of the aggression, not allowing the victim the possibility of survival. It is emphasized that the increase in the use of firearms verified contributes to the increase of the "efficiency" of the practice of the violence. 

Regarding the geographical characteristic, it is worth emphasizing that the violent events follow the municipality's population distribution, with the highest concentration in the urban area. The urban agglomerations are referred to by some authors as a predisposing or facilitating factor for the occurrence of this phenomenon^(^ 1 ^,^ 16 ^)^. The 12 neighborhoods where the highest values were ascertained for the occurrence of acts of violence are found in the outskirts of the city**, localities which are generally less served by social services and amenities. The high rates of homicide, principally of the young, low-income population, are related to the process of unplanned urbanization, socioeconomic inequalities, and poverty^(^ 17 ^)^.

It is possible that the high occurrence of homicides in Itabuna has been contributed to by the crisis in the cocoa industry, which caused intense migratory flow of rural workers and their families from the cocoa bean plantations of the region to the city of Itabuna, principally to the outskirts of the urban area. This phenomenon contributed to an unplanned urbanization, with disorganized populational growth and an offering of goods and community services which was inadequate for this growth. Authors^(^ 5 ^,^ 16 ^)^ point to the significant impacts of the crisis in growing cocoa beans on the generation of income, on the commercial sector, and on unemployment - and on a consequent migratory process. This predominantly rural crisis had direct influences on the urban zone. The populational and urban growth, in convergence with the economic crisis, led to high unemployment and the proliferation of poverty, social exclusion and criminality in the city^(^ 5 ^,^ 18 ^)^.

## Conclusion

The increase in violent criminality in areas of the interior of Brazil, such as in Itabuna, has caused the images and descriptions of urban violence to invade the routine of people in their homes, schools, workplaces and leisure environments. The feeling of neighborliness which characterized residential areas and which was always one of the strong points of the sociability in the interior has been lost: the autonomy, the liberty and spontaneity which characterized mutual recognition and the feeling of closeness, have been profoundly impaired. 

To combat this ubiquitous scourge which tears apart the social fabric and threatens the life, health and happiness of all requires multifocal and intersectorial measures, and actions involving the individual, the family, social groups, the government and the private sector; that is, all the components of society. However, it is necessary that this approach to the violence should direct its attention to primary prevention: rather than simply caring for the consequences of the acts of violence, it is necessary to institute epidemiological surveillance of the violence, allowing the observation of its patterns, risk factors and causes, making it possible to plan, implement and assess effective interventions. 
